# Olive oil intake and risk of cardiovascular disease and mortality in the PREDIMED Study

**DOI:** 10.1186/1741-7015-12-78

**Published:** 2014-05-13

**Authors:** Marta Guasch-Ferré, Frank B Hu, Miguel A Martínez-González, Montserrat Fitó, Mònica Bulló, Ramon Estruch, Emilio Ros, Dolores Corella, Javier Recondo, Enrique Gómez-Gracia, Miquel Fiol, José Lapetra, Lluís Serra-Majem, Miguel A Muñoz, Xavier Pintó, Rosa M Lamuela-Raventós, Josep Basora, Pilar Buil-Cosiales, José V Sorlí, Valentina Ruiz-Gutiérrez, J Alfredo Martínez, Jordi Salas-Salvadó

**Affiliations:** 1 Human Nutrition Unit, University Hospital of Sant Joan de Reus, Faculty of Medicine and Health Sciences, IISPV, Rovira i Virgili University, C/ Sant Llorenç 21, Reus, 43201, Spain; 2 CIBERobn, Institute of Health Carlos III, Madrid, Spain; 3 Department of Nutrition, Harvard School of Public Health, Boston, MA, USA; 4 Department of Preventive Medicine and Public Health, University of Navarra, Pamplona, Spain; 5 Cardiovascular Risk and Nutrition (Regicor Study Group), Hospital del Mar Medical Research Institute, Barcelona Biomedical Research Park, Barcelona, Spain; 6 Department of Internal Medicine, August Pi i Sunyer Institute of Biomedical Research (IDIBAPS), Hospital Clinic, University of Barcelona, Barcelona, Spain; 7 Lipid Clinic, Endocrinology and Nutrition Service, IDIBAPS, Hospital Clinic, University of Barcelona, Barcelona, Spain; 8 Department of Preventive Medicine, University of Valencia, Valencia, Spain; 9 Department of Cardiology, University Hospital Araba, Vitoria, Spain; 10 Department of Preventive Medicine, University of Malaga, Malaga, Spain; 11 Institute of Health Sciences, University of Balearic Islands and Son Espases Hospital, Palma de Mallorca, Spain; 12 Department of Family Medicine, Primary Care Division of Sevilla, San Pablo Health Center, Sevilla, Spain; 13 Research Institute of Biomedical and Health Sciences, University of Las Palmas de Gran Canaria, Las Palmas, Spain; 14 Primary Care Division, Catalan Institute of Health, IDiap-Jordi Gol, Barcelona, Spain; 15 Lipid and Vascular Risk Unit, Department of Internal Medicine, Bellvitge University Hospital, Hospitalet de Llobregat, FIPEC, Barcelona, Spain; 16 Nutrition and Food Safety Research Institute, Faculty of Pharmacy, University of Barcelona, Barcelona, Spain; 17 Primary care, Servicio Navarro de Salud Osadunbidea, Pamplona, Spain; 18 La Grasa Institute, Spanish National Research Council, Sevilla, Spain; 19 Department of Food Science Nutrition, Physiology and Toxicology, University of Navarra, Pamplona, Spain

**Keywords:** Olive oil, Cardiovascular, Mortality, Mediterranean Diet, PREDIMED

## Abstract

**Background:**

It is unknown whether individuals at high cardiovascular risk sustain a benefit in cardiovascular disease from increased olive oil consumption. The aim was to assess the association between total olive oil intake, its varieties (extra virgin and common olive oil) and the risk of cardiovascular disease and mortality in a Mediterranean population at high cardiovascular risk.

**Methods:**

We included 7,216 men and women at high cardiovascular risk, aged 55 to 80 years, from the PREvención con DIeta MEDiterránea (PREDIMED) study, a multicenter, randomized, controlled, clinical trial. Participants were randomized to one of three interventions: Mediterranean Diets supplemented with nuts or extra-virgin olive oil, or a control low-fat diet. The present analysis was conducted as an observational prospective cohort study. The median follow-up was 4.8 years. Cardiovascular disease (stroke, myocardial infarction and cardiovascular death) and mortality were ascertained by medical records and National Death Index. Olive oil consumption was evaluated with validated food frequency questionnaires. Multivariate Cox proportional hazards and generalized estimating equations were used to assess the association between baseline and yearly repeated measurements of olive oil intake, cardiovascular disease and mortality.

**Results:**

During follow-up, 277 cardiovascular events and 323 deaths occurred. Participants in the highest energy-adjusted tertile of baseline total olive oil and extra-virgin olive oil consumption had 35% (HR: 0.65; 95% CI: 0.47 to 0.89) and 39% (HR: 0.61; 95% CI: 0.44 to 0.85) cardiovascular disease risk reduction, respectively, compared to the reference. Higher baseline total olive oil consumption was associated with 48% (HR: 0.52; 95% CI: 0.29 to 0.93) reduced risk of cardiovascular mortality. For each 10 g/d increase in extra-virgin olive oil consumption, cardiovascular disease and mortality risk decreased by 10% and 7%, respectively. No significant associations were found for cancer and all-cause mortality. The associations between cardiovascular events and extra virgin olive oil intake were significant in the Mediterranean diet intervention groups and not in the control group.

**Conclusions:**

Olive oil consumption, specifically the extra-virgin variety, is associated with reduced risks of cardiovascular disease and mortality in individuals at high cardiovascular risk.

**Trial registration:**

This study was registered at controlled-trials.com (http://www.controlled-trials.com/ISRCTN35739639). International Standard Randomized Controlled Trial Number (ISRCTN): 35739639. Registration date: 5 October 2005.

## Background

Olive oil is a key component of the Mediterranean Diet (MedDiet), being the main source of vegetable fat, especially monounsaturated fatty acids (MUFA) [1]. Virgin olive oil, produced by mechanically pressing ripe olives, contains multiple bioactive and antioxidant components such as polyphenols, phytosterols and vitamin E [1], and has an acidity of <1.5%. Extra-virgin olive oil (EVOO) is also produced by mechanically pressing the olives but is the oil with the best quality, the most intense taste and its acidity is <1%. In contrast, common olive oil, obtained from a mixture of virgin and refined oil (usually more than 80% is refined), has fewer antioxidant and anti-inflammatory compounds. Since refined olive oil during the refining process loses phytochemicals, this oil is mixed with virgin olive oil to enhance the flavor, constituting the so-called common olive oil [2].

Evidence suggests that olive oil intake is inversely associated with cardiovascular disease (CVD) in the Spanish general population [3] and in a cohort of Italian women [4]. In the Spanish cohort of the European Prospective Investigation into Cancer and Nutrition (EPIC) study, total olive oil intake has been associated with a decreased risk of coronary heart disease, and also all-cause and cardiovascular mortality [5,6]. Similarly, a lower risk of mortality was associated with regular consumption of olive oil in an Italian population after myocardial infarction [7] and also in an elderly population [8]. A recent meta-analysis concluded that epidemiologic studies consistently found an inverse association between olive oil consumption and stroke, but there were inconsistencies between studies assessing coronary heart disease (CHD) as the end-point [9]. Of note, most of the previous studies made no distinction among the different varieties of olive oil [4,7,8]. Except for the EPIC-Spanish cohort that found a greater beneficial effect in CHD for the virgin olive oil variety than for the common variety [5] and similar effects for both varieties on all-cause mortality [6]. This distinction is important because EVOO contains much higher amounts of polyphenols than common olive oil. These polyphenols may have cardiovascular benefits beyond the lipid profile. It has also been reported that olive oil intake could be beneficial in the prevention of certain cancers, such as breast cancer [10], but the evidence is weaker.

Recently, in the context of the PREvención con DIeta MEDiterránea (PREDIMED) Study, it has been demonstrated that a MedDiet enriched with EVOO improved lipid profile, decreased blood pressure and reduced the risk of major cardiovascular events [11,12]. In this observational analysis of the PREDIMED population, we aimed to assess the associations between baseline olive oil consumption and the risk of CVD, cause-specific and overall mortality. We hypothesized that higher consumption of olive oil, especially the EVOO variety, would be associated with a decreased risk of CVD, cause-specific and overall mortality in an elderly Mediterranean population at high cardiovascular risk, independent of the allocated arm of the trial.

## Methods

### Study population

The present study was conducted within the framework of the PREDIMED trial, whose design has been described in detail elsewhere [13]. Briefly, the PREDIMED study is a large, multicenter, parallel-group, randomized and controlled clinical trial for the primary prevention of CVD (http://www.predimed.es). The main results of the trial on the primary endpoint have been published elsewhere [12]. We assigned 7,447 participants (men aged 55 to 80 years and women 60 to 80 years) to one of three interventions: a MedDiet supplemented with EVOO, a MedDiet supplemented with mixed nuts, or advice on a low-fat diet (control diet). Participants had no CVD at enrollment, but they were at high cardiovascular risk because of the presence of type 2 diabetes or at least three of the following risk factors: current smoking, hypertension, high low-density lipoprotein (LDL)-cholesterol, low high-density lipoprotein (HDL)-cholesterol, overweight or obesity, and family history of premature CVD. Exclusion criteria were the presence of any severe chronic illness, alcohol or drug abuse, body mass index (BMI) ≥40 kg/m^2^, and allergy or intolerance to olive oil or nuts. The primary endpoint of the main trial was a composite of cardiovascular events (myocardial infarction, stroke or death from cardiovascular causes). The present analysis was conducted as an observational prospective cohort study using baseline consumption of olive oil as the exposure, and taking baseline data from the date of the first visit, before the individuals were randomized to the intervention group. The outcomes were: (a) composite of cardiovascular events, (b) cardiovascular mortality, (c) cancer mortality and (d) all-cause mortality. All participants provided written informed consent according to a protocol approved by the institutional review boards of all the recruiting centers (Comité de Ética e Investigación Clínica [CEIC] Hospital Universitari Sant Joan de Reus, CEIC Universidad de Navarra, CEIC Hospital Clínic de Barcelona, Comité de Ética Universidad de Valencia, CEIC-Parc de Salut Mar, CEIC Hospital Universitario Araba, CEIS del distrito Sanitario Atención Primaria Sevilla, IDIAP Jordi Gol, CEIC Complejo Hospitalario Materno-Insular, CEIC Facultad Medicina Universidad de Málaga, CEIC Illes Balears, CEIC Hospital Universitari Bellvitge).

### Assessment of olive oil consumption and other covariates

At baseline and yearly during the follow-up, trained dieticians completed a 137-item semiquantitative food frequency questionnaire (FFQ) in a face-to-face interview with the participants. This questionnaire has been validated before in a population at high cardiovascular risk from Spain. Reproducibility and validity of the FFQ for total olive oil consumption, estimates by the Pearson correlation coefficient (r) were 0.55 and 0.60, respectively, and the intraclass correlation coefficients for reproducibility and validity were 0.71 (*P* <0.001) [14]. Energy and nutrient intake were estimated using updated Spanish food composition tables [15,16]. Information on consumption of different types of olive oil intake was derived from the FFQ. The questionnaire includes three different questions regarding the consumption of olive oil: EVOO intake (produced by mechanically pressing the olives, acidity <1%), refined olive oil intake (refined olive oil, acidity <0.3%) and pomace olive oil (obtained from the residue of pressing the olives and mixed with other refined olive oils, acidity <0.3%). The dieticians asked the participants if they consumed one tablespoon of olive oil (for each of the specific varieties): never, between one to three times per month, times per week (once, two to four, five to six, three options) or times a day (once, two to three, four to six, more than six, four options). These questions were transformed to continuous variables in grams per day. The first question was used to assess EVOO intake, the sum of the second and third questions (refined olive oil and pomace olive oil) was considered common olive oil intake. The sum of all the three questions provides the total amount of olive oil consumed.

In addition, dieticians administered a validated 14-item MedDiet screener designed to assess the degree of adherence to the traditional MedDiet [17]. Two questions in the screener are related to olive oil intake (use of olive oil as the main fat for cooking (1 point if the answer is yes) and using four or more tablespoons of olive oil (1 point if the answer is yes), with 14 points the total score of the questionnaire). To control for the overall dietary pattern, we used this MedDiet screener removing the variables related to olive oil consumption; thus, a 12-point score was used as a covariate in the models.

At baseline, a questionnaire about lifestyle variables, educational achievement, history of illnesses and medication use was administered. Physical activity was assessed using the validated Spanish version of the Minnesota Leisure-Time Physical Activity questionnaire [18]. Participants were considered to be diabetic, hypercholesterolemic or hypertensive if they had previously been diagnosed as such, and/or they were being treated with antidiabetic, cholesterol-lowering or antihypertensive agents, respectively. Trained personnel took the anthropometric and blood pressure measurements. Weight and height were measured with light clothing and no shoes with calibrated scales and a wall-mounted stadiometer, respectively. Waist circumference was measured midway between the lowest rib and the iliac crest using an anthropometric tape. Blood pressure was measured using a validated oscillometer (Omron HEM705CP; Hoofddorp, The Netherlands) in triplicate with a five-minute interval between each measurement, and the mean of these values was recorded.

### Ascertainment of cardiovascular disease and mortality

Information on CVD and mortality was updated once a year by the End-point Adjudication Committee, whose members were blinded to treatment allocation and to the dietary habits of participants. Different sources of information were used: (a) yearly questionnaires and examinations for all participants, (b) primary care physicians, (c) yearly review of medical records, and (d) linkage to the National Death Index. Medical records of deceased participants were requested. The End-point Adjudication Committee adjudicated the cause of the death and confirmed cardiovascular events.

### Statistical analyses

Follow-up time was calculated as the interval between the date of cardiovascular events, death (cardiovascular, cancer or all-causes of death) or the end of follow-up (the date of the last visit or the last recorded clinical event of participants still alive) and the date of randomization. Extremes of reported total energy intake (>4,000 or <800 kcal per day in men and >3,500 or <500 kcal per day in women) were excluded from the present analysis [19]. Baseline characteristics of the studied population are presented according to energy-adjusted tertiles of total olive oil consumption, as means (SD) for quantitative variables and percentage (number) for categorical variables.

Multivariate Cox proportional hazard models were used to assess the associations between baseline energy-adjusted tertiles of olive oil intake and the risk of CVD, cardiovascular mortality, cancer mortality and all-cause mortality. Baseline total olive oil intake has also been analyzed as a continuous variable. All analyses were repeated using energy-adjusted tertiles of EVOO and common olive oil consumption. We also tested the associations between baseline olive fruit consumption and CVD and mortality (consumption of the whole olive fruit, not oil). All analyses were stratified by the recruitment center. Results are expressed as hazard ratios (HRs) with 95% confidence intervals (CIs). Model 1 was adjusted for age (continuous), sex and the intervention group. Model 2 was additionally adjusted for BMI (kg/m^2^), smoking status (never, former, current smoker), alcohol intake (continuous, adding a quadratic term), educational level (illiterate/primary education, secondary education, academic/graduate), leisure time physical activity (Metabolic Equivalent of Task (MET)-minutes/d), prevalence of diabetes (yes/no), prevalence of hypertension (yes/no), prevalence of hypercholesterolemia (yes/no), use of antihypertensive medication (yes/no) and use of statins (yes/no). A third model was additionally adjusted for baseline MedDiet adherence (12-point score). Additionally, myocardial infarction and stroke were used as outcomes of the analysis. Linear trend tests were assessed using the median value to each category of total olive oil, EVOO and common olive oil consumption and included as a continuous variable in the various models. We used Cox regression models to assess the association between total olive oil and CVD, cardiovascular mortality, cancer mortality and all-cause mortality separated by intervention group. Linear trends were also tested. As a secondary analysis and to take advantage of the updated information of yearly intake of total olive oil, we repeated the analysis using generalized estimating equations to assess the association between yearly repeated measurements of total olive oil consumption during follow-up and CVD and mortality. For each one-year period we used as exposure the average of total olive oil consumption of all repeated measurements from baseline to the beginning of that yearly period (between two and eight years). Sensitivity analyses were conducted including only the events observed in the two first years, between the second and fourth years, and after four years of follow-up. Also, sensitivity analyses were conducted excluding early cases (less than one year) and late cases (more than four years). The level of significance for all statistical tests was *P*<0.05 for bilateral contrast. Analyses were done using SPSS statistical software, version 19 (SPSS Inc., Chicago, IL, USA).

## Results

After excluding those individuals with extremes of reported total energy intake (n = 153) and those with incomplete baseline dietary data (n = 78), a total of 7,216 participants were included in the present analysis. During a median of 4.8 y of follow-up we documented 277 incident cases of major cardiovascular events, 81 cardiovascular deaths, 130 cancer deaths and 323 all-cause deaths. The mean age of the participants was 67 y and 57.4% of them were women, respectively. The baseline characteristics of the participants according to energy-adjusted tertiles of total olive oil consumption are described in Table 1. As compared with participants in the lowest tertile of total olive oil intake, those in the highest tertile were more likely to have secondary education, lower total energy intake, lower consumption of red meat and dairy products, and also to drink less alcohol. The mean intake of total olive oil was 56.9 g/d in those participants allocated in the highest tertile compared to 21.4 g/d in those of the lowest tertile. Changes in total olive oil intake at the end of follow-up were 3.85 ± 23.02 g/d (mean ± SD) for the combined participants, 10.92 ± 22.91 g/d for those given MedDiet supplemented with EVOO, 2.36 ± 21.81 g/d for those given MedDiet supplemented with nuts and -3.03 ± 22.02 g/d in the control group. The total olive oil consumption by year during the follow-up for the total participants, and also by the intervention group is shown in Additional file 1.

**Table 1:** Baseline characteristics of study participants according to energy-adjusted tertiles of total olive oil consumption

**Baseline energy-adjusted tertiles of total olive oil consumption**				
**Variable**	**1 (n = 2,405)**	**2 (n = 2,406)**	**3 (n = 2,405)**	***P*-value**
**Age (y)**	67 ± 6	67 ± 6	67 ± 6	0.744
**Men, % (n)**	45.7 (1,099)	42.2 (1,016)	39.8 (956)	<0.001
**Intervention group, % (n)**				0.476
**MedDiet + EVOO**	33.1 (796)	34.5 (830)	35.3 (848)	
**MedDiet + Nuts**	32.6 (785)	32.9 (792)	32.6 (783)	
**Control low fat diet**	34.3 (824)	32.6 (784)	32.2 (774)	
**BMI (kg/m**^**2**^**)**	29.9 ± 3.73	29.9 ± 3.88	30.0 ± 3.93	0.427
**Weight (kg)**	77.0 ± 11.7	76.6 ± 12.0	76.6 ± 12.1	0.422
**Leisure-time energy expenditure in physical activity (MET minutes/d)**	243.1 ± 265.2	228.6 ± 225.5	221.3 ± 223.1	0.005
**Smoking status, % (n)**				0.262
**Never**	60.3 (1,450)	60.9 (1,466)	63.3 (1,523)	
**Current**	14.4 (347)	14.2 (341)	13.1 (316)	
**Former**	25.3 (608)	24.9 (599)	23.5 (566)	
**Educational level, % (n)**				0.040
**Illiterate/primary education**	79.3 (1,907)	77.6 (1,866)	76.1 (1,831)	
**Secondary education**	14.1 (338)	14.7 (354)	16.8 (404)	
**Academic/graduate**	6.7 (160)	7.7 (186)	7.1 (170)	
**Prevalence of diabetes, % (n)**	49.9 (1,200)	47.9 (1,153)	48.1 (1,156)	0.312
**Prevalence of hypertension, % (n)**	83.1 (1,998)	82.1 (1,976)	83.0 (1,995)	0.639
**Prevalence of hypercholesterolemia, % (n)**	73.6 (1,769)	71.7 (1,726)	71.4 (1,716)	0.190
**Family history of coronary heart disease, % (n)**	22.3 (536)	22.1 (531)	22.6 (544)	0.899
**Medication use, % (n)**				
**Oral antidiabetic drugs**	33.6 (808)	31.4 (756)	31.6 (759)	0.195
**Antihypertensive drugs**	71.9 (1,728)	72.4 (1,743)	73.9 (1,777)	0.264
**Statins**	40.4 (972)	40.5 (974)	39.8 (958)	0.880
**Modified MedDiet score (12-point score)**	6.9 ± 1.7	7.0 ± 1.7	7.2 ± 1.7	<0.001
**Total energy intake (kcal/d)**	2,266 ± 479	2,242 ± 668	2,199 ± 457	<0.001
**Total olive oil (g/d)**	21.4 ± 8.00	38.8 ± 11.6	56.9 ± 10.8	<0.001
**Extra virgin olive oil (g/d)**	9.1 ± 11.2	19.5 ± 20.0	34.6 ± 27.4	<0.001
**Common olive oil (g/d)**	12.1 ± 11.7	18.6 ± 18.5	21.7 ± 25.9	<0.001
**Alcohol (g/d)**	8.51 ± 14.1	9.41 ± 15.5	7.06 ± 12.2	<0.001
**Nuts (g/d)**	11.3 ± 14.8	10.2 ± 14.2	8.7 ± 11.5	<0.001
**Vegetables (g/d)**	346 ± 156	328 ± 145	327 ± 137	<0.001
**Fruit (g/d)**	389 ± 219	363 ± 196	351 ± 184	<0.001
**Red meat (beef, pork, lamb) (g/d)**	79.6 ± 49.1	79.1 ± 46.1	70.4 ± 41.2	<0.001
**White meat (chicken, rabbit, turkey) (g/d)**	46.4 ± 28.2	45.9 ± 27.6	42.1 ± 27.1	<0.001
**Eggs (g/d)**	20.5 ± 10.2	20.1 ± 11.5	19.3 ± 11.5	<0.001
**Fish (g/d)**	95.7 ± 51.9	99.8 ± 52.0	102 ± 46.9	<0.001
**Dairy (g/d)**	407 ± 231	375 ± 220	357 ± 205	<0.001

Data are expressed as means ± SD or percentage (n). *P*-value for comparisons across tertiles of baseline energy-adjusted olive oil consumption (Pearson chi-square test for categorical variables or 1-way analysis of variance for continuous variable) as appropriate. BMI, body mass index; EVOO, extra virgin olive oil; MedDiet, Mediterranean diet; MET, Metabolic Equivalent of Task.

After adjusting for age, sex and intervention group, there was a 34% (*P* for trend = 0.01) lower risk of major cardiovascular events among individuals in the highest tertile of baseline total olive oil intake as compared to those in the reference (Table 2). The inverse association remained statistically significant (*P* for trend = 0.01) after the adjustment for lifestyle variables and other potential confounders (36% lower risk (HR: 0.64; 95% CI: 0.46 to 0.87)) and even after further adjustment for MedDiet adherence (35% lower risk (HR: 0.65; 95% CI: 0.47 to 0.89)). For each 10 g/d (one tablespoon) higher baseline total olive oil consumption there was a 13% (HR: 0.87; 95% CI: 0.81 to 0.94) decreased risk of major cardiovascular events. A 48% reduction in the risk of cardiovascular death (HR: 0.52; 95% CI: 0.29 to 0.93) was found in the fully-adjusted model for those individuals in the highest tertile of total baseline olive oil consumption as compared to the reference. Also, for each 10 g/d (one tablespoon) higher total baseline olive oil consumption there was a 16% (HR: 0.84; 95% CI: 0.73 to 0.96) decreased risk of cardiovascular mortality. No statistically significant associations were found for cancer mortality and all-cause mortality.

**Table 2:** Risk of cardiovascular events and mortality according to baseline total olive oil intake

	**Energy-adjusted tertiles of total olive oil, g/day**				
	**1 (low) (n = 2,405)**	**2 (n = 2,406)**	**3 (high) (n = 2,405)**	***P for trend***	**Energy-adjusted total olive oil intake (10 g/d)**
**Mean total olive oil intake**	**21.4 ± 8.00**	**38.8 ± 11.6**	**56.9 ± 10.8**		
**Major event**					
Cardiovascular event, % (n)	4.5 (108)	3.6 (86)	3.5 (83)		3.8 (277)
Multivariable model 1	1 (Ref.)	0.76 (0.57, 1.02)	0.66 (0.48, 0.90)	0.01	0.87 (0.81, 0.94)
Multivariable model 2	1 (Ref.)	0.78 (0.58, 1.04)	0.64 (0.46, 0.87)	0.01	0.87 (0.81, 0.94)
Multivariable model 3	1 (Ref.)	0.78 (0.58, 1.04)	0.65 (0.47, 0.89)	0.01	0.87 (0.81, 0.94)
**Cardiovascular mortality**	**1 (low) (n = 2,405)**	**2 (n = 2,406)**	**3 (high) (n = 2,405)**	***P for trend***	
Cardiovascular mortality, % (n)	1.4 (33)	1.0 (25)	1.0 (23)		1.1 (81)
Multivariable model 1	1 (Ref.)	0.68 (0.39, 1.16)	0.52 (0.29, 0.94)	0.04	0.83 (0.72, 0.96)
Multivariable model 2	1 (Ref.)	0.70 (0.41, 1.20)	0.51 (0.28, 0.92)	0.04	0.83 (0.72, 0.95)
Multivariable model 3	1 (Ref.)	0.69 (0.40, 1.18)	0.52 (0.29, 0.93)	0.04	0.84 (0.73, 0.96)
**Cancer mortality**	**1 (low) (n = 2,405)**	**2 (n = 2,406)**	**3 (high) (n = 2,405)**	***P for trend***	
Cancer mortality, % (n)	1.8 (44)	2.0 (49)	1.5 (37)		1.8 (130)
Multivariable model 1	1 (Ref.)	1.13 (0.74, 1.72)	0.80 (0.49, 1.30)	0.96	0.93 (0.83, 1.05)
Multivariable model 2	1 (Ref.)	1.13 (0.74, 1.72)	0.84 (0.52, 1.36)	0.95	0.95 (0.84, 1.07)
Multivariable model 3	1 (Ref.)	1.13 (0.74, 1.72)	0.84 (0.52, 1.37)	0.94	0.95 (0.85, 1.07)
**All-cause mortality**	**1 (low) (n = 2,405)**	**2 (n = 2,406)**	**3 (high) (n = 2,405)**	***P for trend***	
All causes of mortality, % (n)	4.8 (116)	4.4 (106)	4.2 (101)		4.5 (323)
Multivariable model 1	1 (Ref.)	0.90 (0.69, 1.19)	0.79 (0.59, 1.06)	0.21	0.93 (0.87, 1.00)
Multivariable model 2	1 (Ref.)	0.90 (0.69, 1.18)	0.77 (0.58, 1.04)	0.18	0.93 (0.87, 1.00)
Multivariable model 3	1 (Ref.)	0.90 (0.69, 1.18)	0.78 (0.58, 1.05)	0.18	0.94 (0.87, 1.00)

Cox regression models were used to assess the risk of cardiovascular events and mortality by baseline energy-adjusted tertiles of total olive oil (g/day) and as a continuous variable (10 g/d). Results were presented as Hazard Ratios (95% CI). Multivariable model 1 was adjusted for age (years), sex and theintervention group. Model 2 was also adjusted for body mass index (BMI) (kg/m^2^), smoking status (never, former, current smoker), alcohol intake (continuous, adding a quadratic term), educational level (illiterate/primary education, secondary education, academic/graduate), leisure time physical activity (Metabolic Equivalent of Task (MET)-minutes/d), prevalence of diabetes (yes/no), prevalence of hypertension (yes/no), prevalence of hypercholesterolemia (yes/no), use of antihypertensive medication (yes/no) and use of statins (yes/no). Model 3 was also adjusted for Mediterranean diet adherence (Modified 12-point Mediterranean Diet score). All models were stratified by recruitment center. Extremes of total energy intake were excluded. A major event was a composite of myocardial infarction, stroke and death from cardiovascular causes.

The HR and 95% CIs for the association between EVOO, CVD and also for mortality are presented in Table 3. Baseline EVOO consumption was inversely associated with major events after adjusting for potential confounders (39% lower risk (HR: 0.61; 95% CI: 0.44 to 0.85 (*P* for trend <0.01)). A non-significant inverse association between baseline EVOO consumption and mortality outcomes was found, specifically for overall mortality. We observed non-significant associations between the baseline intake of common olive oil and major events and mortality (Table 4).

**Table 3:** Risk of cardiovascular events and mortality according to baseline extra-virgin olive oil intake

	**Energy-adjusted tertiles of extra-virgin olive oil, g/day**				
	**1 (low) (n = 2,405)**	**2 (n = 2,406)**	**3 (high) (n = 2,405)**	***P for trend***	**Energy-adjusted extra virgin olive oil intake (10 g/d)**
**Mean extra-virgin olive oil intake**	**9.1 ± 11.23**	**19.5 ± 20.0**	**34.6 ± 27.4**		
**Major event**					
Cardiovascular event, % (n)	4.6 (111)	4.2 (101)	2.7 (65)		3.8 (277)
Multivariable model 1	1 (Ref.)	1.01 (0.77, 1.33)	0.60 (0.43, 0.82)	< 0.01	0.89 (0.84, 0.95)
Multivariable model 2	1 (Ref.)	1.00 (0.76, 1.32)	0.60 (0.44, 0.84)	< 0.01	0.90 (0.85, 0.95)
Multivariable model 3	1 (Ref.)	0.99 (0.75, 1.31)	0.61 (0.44, 0.85)	< 0.01	0.90 (0.85, 0.95)
**Cardiovascular mortality**	**1 (low) (n = 2,405)**	**2 (n = 2,406)**	**3 (high) (n = 2,405)**	***P for trend***	
Cardiovascular mortality, % (n)	1.3 (32)	1.2 (28)	0.9 (21)		1.1 (81)
Multivariable model 1	1 (Ref.)	1.01 (0.60, 1.70)	0.64 (0.36, 1.15)	0.10	0.93 (0.84, 1.03)
Multivariable model 2	1 (Ref.)	0.99 (0.59, 1.67)	0.64 (0.36, 1.15)	0.10	0.93 (0.83, 1.03)
Multivariable model 3	1 (Ref.)	0.97 (0.58, 1.64)	0.65 (0.36, 1.17)	0.13	0.93 (0.84, 1.03)
**Cancer mortality**	**1 (low) (n = 2,405)**	**2 (n = 2,406)**	**3 (high) (n = 2,405)**	***P for trend***	
Cancer mortality, % (n)	2.1 (50)	1.7 (41)	1.6 (39)		1.8 (130)
Multivariable model 1	1 (Ref.)	0.90 (0.59, 1.37)	0.87 (0.56, 1.37)	0.61	0.96 (0.88, 1.04)
Multivariable model 2	1 (Ref.)	0.88 (0.58, 1.35)	0.88 (0.56, 1.39)	0.68	0.96 (0.89, 1.05)
Multivariable model 3	1 (Ref.)	0.89 (0.58, 1.35)	0.90 (0.57, 1.41)	0.73	0.97 (0.89, 1.05)
**All-cause mortality**	**1 (low) (n = 2,405)**	**2 (n = 2,406)**	**3 (high) (n = 2,405)**	***P for trend***	
All causes of mortality, % (n)	5.2 (125)	4.2 (100)	4.1 (98)		4.5 (323)
Multivariable model 1	1 (Ref.)	0.88 (0.67, 1.15)	0.81 (0.61, 1.07)	0.19	0.95 (0.91, 1.00)
Multivariable model 2	1 (Ref.)	0.84 (0.64, 1.10)	0.80 (0.60, 1.07)	0.20	0.95 (0.90, 1.00)
Multivariable model 3	1 (Ref.)	0.84 (0.64, 1.10)	0.82 (0.61, 1.09)	0.25	0.96 (0.91, 1.01)

Cox regression models were used to assess the risk of cardiovascular events and mortality by baseline energy-adjusted tertiles of extra virgin olive oil (g/day) and as a continuous variable (10 g/d). Results were presented as Hazard Ratios (95% CI). Multivariable model 1 was adjusted for age (years), sex and the intervention group. Model 2 was also adjusted for body mass index (BMI) (kg/m^2^), smoking status (never, former, current smoker), alcohol intake (continuous, adding a quadratic term), educational level (illiterate/primary education, secondary education, academic/graduate), leisure time physical activity (Metabolic Equivalent of Task (MET)-minutes/d), prevalence of diabetes (yes/no), prevalence of hypertension (yes/no), prevalence of hypercholesterolemia (yes/no), use of antihypertensive medication (yes/no) and use of statins (yes/no). Model 3 was also adjusted for Mediterranean diet adherence (Modified 12-point Mediterranean Diet score). All models were stratified by recruitment center. Extremes of total energy intake were excluded. A major event was a composite of myocardial infarction, stroke and death from cardiovascular causes.

**Table 4:** Risk of cardiovascular events and mortality according to baseline common olive oil intake

	**Energy-adjusted tertiles of common olive oil, g/day**				
	**1 (low) (n = 2,405)**	**2 (n = 2,406)**	**3 (high) (n = 2,405)**	***P for trend***	**Energy-adjusted common olive oil intake (10 g/d)**
**Mean common olive oil intake**	**12.1 ± 11.7**	**18.6 ± 18.5**	**21.7 ± 25.9**		
**Major event**					
Cardiovascular event, % (n)	3.5 (85)	3.6 (86)	4.4 (106)		3.8 (277)
Multivariable model 1	1 (Ref.)	1.06 (0.78, 1.45)	1.20 (0.88, 1.62)	0.23	1.04 (0.99, 1.10)
Multivariable model 2	1 (Ref.)	1.01 (0.74, 1.38)	1.13 (0.83, 1.54)	0.35	1.04 (0.98, 1.10)
Multivariable model 3	1 (Ref.)	0.99 (0.73, 1.36)	1.11 (0.82, 1.51)	0.40	1.03 (0.98, 1.09)
**Cardiovascular mortality**	**1 (low) (n = 2,405)**	**2 (n = 2,406)**	**3 (high) (n = 2,405)**	***P for trend***	
Cardiovascular mortality, % (n)	1.3 (31)	1.0 (24)	1.1 (26)		1.1 (81)
Multivariable model 1	1 (Ref.)	0.87 (0.50, 1.51)	0.84 (0.48, 1.46)	0.60	0.98 (0.88, 1.09)
Multivariable model 2	1 (Ref.)	0.84 (0.48, 1.47)	0.84 (0.48, 1.48)	0.65	0.98 (0.88, 1.09)
Multivariable model 3	1 (Ref.)	0.83 (0.47, 1.46)	0.81 (0.46, 1.43)	0.56	0.98 (0.87, 1.09)
**Cancer mortality**	**1 (low) (n = 2,405)**	**2 (n = 2,406)**	**3 (high) (n = 2,405)**	***P for trend***	
Cancer mortality, % (n)	1.7 (40)	1.7 (42)	2.0 (48)		1.8 (130)
Multivariable model 1	1 (Ref.)	1.07 (0.68, 1.68)	1.16 (0.74, 1.82)	0.51	1.01 (0.92, 1.10)
Multivariable model 2	1 (Ref.)	1.04 (0.66, 1.64)	1.16 (0.74, 1.82)	0.49	1.01 (0.93, 1.10)
Multivariable model 3	1 (Ref.)	1.03 (0.65, 1.62)	1.14 (0.73, 1.79)	0.52	1.01 (0.92, 1.10)
**All-cause mortality**	**1 (low) (n = 2,405)**	**2 (n = 2,406)**	**3 (high) (n = 2,405)**	***P for trend***	
All causes of mortality, % (n)	4.2 (101)	4.4 (106)	4.8 (116)		4.5 (323)
Multivariable model 1	1 (Ref.)	1.14 (0.86, 1.51)	1.17 (0.88, 1.51)	0.34	1.01 (0.96, 1.07)
Multivariable model 2	1 (Ref.)	1.10 (0.83, 1.47)	1.16 (0.87, 1.54)	0.37	1.01 (0.96, 1.07)
Multivariable model 3	1 (Ref.)	1.09 (0.82, 1.45)	1.14 (0.85, 1.51)	0.44	1.01 (0.96, 1.07)

Cox regression models were used to assess the risk of cardiovascular events and mortality by baseline energy-adjusted tertiles of common olive oil (g/day) and as a continuous variable (10 g/d). Results were presented as Hazard Ratios (95% CI). Multivariable model 1 was adjusted for age (years), sex and the intervention group. Model 2 was also adjusted for body mass index (BMI) (kg/m^2^), smoking status (never, former, current smoker), alcohol intake (continuous, adding a quadratic term), educational level (illiterate/primary education, secondary education, academic/graduate), leisure time physical activity (Metabolic Equivalent of Task (MET)-minutes/d), prevalence of diabetes (yes/no), prevalence of hypertension (yes/no), prevalence of hypercholesterolemia (yes/no), use of antihypertensive medication (yes/no) and use of statins (yes/no). Model 3 was also adjusted for Mediterranean diet adherence (Modified 12-point Mediterranean Diet score). All models were stratified by recruitment center. Extremes of total energy intake were excluded. A major event was a composite of myocardial infarction, stroke and death from cardiovascular causes.

When we screened the risk of myocardial infarction and stroke according to the different categories and varieties of olive oil consumption it was observed that the inverse associations were non-statistically significant (data not shown).

We have also analyzed the association between the consumption of olive fruit (olives) and the risk of major events. We found an association of 25% decrease in the risk of major events in the top tertile of baseline olive fruit consumption after adjusting for potential confounders (HR: 0.75; 95% CI: 0.55 to 1.01, *P* for trend = 0.10).

The reduction in the risk of major cardiovascular events according to tertiles of total baseline olive oil intake separated by intervention group were 57% (HR: 0.43; 95% CI: 0.25 to 0.75, *P* for trend <0.01) and 55% (HR: 0.45; 95% CI: 0.25 to 0.82, *P* for trend <0.01) in the groups of MedDiet supplemented with EVOO or nuts, respectively. In contrast, the risk in the low fat control group was increased by 9% (HR: 1.09, 95% CI: 0.63 to 1.88, *P* for trend = 0.24) (*P-*value of homogeneity test: 0.178). The association between major events and EVOO intake showed relative risk reductions of 41% (HR: 0.59; 95% CI: 0.32 to 1.07, *P* for trend = 0.050), 63% (HR: 0.37; 95% CI: 0.20 to 0.71, *P* for trend <0.01) and 15% (HR: 0.85; 95% CI: 0.51 to 1.41, *P* for trend = 0.503) in the MedDiet supplemented with EVOO, nuts and control group, respectively (*P-*value of homogeneity test: 0.364).

The inverse association between yearly updated measurements of total olive oil consumption and CVD using generalized estimating equations were also statistically significant after adjusting for potential confounders. The fully adjusted relative risk (RR) in the highest tertile of total olive oil consumption, as compared to the reference, showed a relative risk reduction of 34% (HR: 0.66; 95% CI: 0.48 to 0.91) with a significant linear trend test (*P* for trend <0.01). When we repeated the analysis to evaluate the associations between total olive oil consumption and cardiovascular mortality and cancer mortality, the fully adjusted relative risk (RR) in the top tertile of total olive oil showed a relative risk reduction of 44% (HR: 0.56; 95% CI: 0.31 to 1.02) and 24% (HR: 0.76; 95% CI: 0.46 to 1.24), respectively. However, the linear trend tests were non-significant. Finally, the fully adjusted relative risk (RR) in the top tertile of total olive oil consumption showed a relative risk reduction of 25% (HR: 0.75; 95% CI: 0.56 to 1.00, *P* for trend <0.01) for all-cause mortality.

The results of several sensitivity analyses were consistent with the findings of the primary analysis. When only the events observed in the first two years (91 events included) were considered, the risk of a major event in the top tertile of total olive oil was: 0.87 (95% CI: 0.50 to 1.51). When only events between the second and fourth years (99 events) were considered, the RR in the top tertile of olive oil was: 0.55 (95% CI: 0.33 to 0.93), and only including events occurred after four years the RR was: 0.56 (95% CI: 0.31 to 1.01). The RR for the top tertile of olive oil excluding early cases occurred in the first year (230 events included) was 0.60 (95% CI: 0.43 to 0.85), and excluding late cases after four years (190 events included) the RR was 0.68 (95% CI: 0.46 to 0.98).

## Discussion

In this prospective study of Mediterranean individuals at high cardiovascular risk, we found that baseline total olive oil consumption, especially the extra-virgin variety, was associated with a significant reduced risk of major cardiovascular events and cardiovascular mortality in a Mediterranean population at high cardiovascular risk. Relative risk reductions in CVD and all-cause mortality were similar for the upper baseline category of total olive oil consumption when we evaluated the repeated measurements of total olive oil consumption over time. We also found a reduction in cardiovascular mortality for an increased consumption of total olive oil. Each increase of 10 g/d in EVOO intake was associated with a 10% reduction in the risk of cardiovascular events. To the contrary, consumption of common olive oil was not significantly associated with cardiovascular morbidity and mortality.

In both MedDiet groups of our study (supplemented either with EVOO or nuts), participants in the top tertile of total olive oil consumption at baseline showed a lower risk of major events compared to those in the lowest tertile, but no associations were found for those individuals allocated to the control group. One explanation could be that the advice against eating fat food such as olive oil in the low fat control group throughout the study might have counterbalanced the protective effect of a lifetime intake of olive oil. However, we found inverse associations between olive oil consumption and CVD not only in the group supplemented with EVOO but also in the nuts group. Although it is difficult to isolate the effect of a single food because a range of foods are consumed in the whole diet, our study was able to distinguish the effects attributed to olive oil, a food that it is clearly a key component of the MedDiet. The positive association found in the MedDiet groups seems to confirm these effects.

Recent findings of the PREDIMED Study have demonstrated that adherence to the Mediterranean dietary pattern, as a whole and enriched with EVOO or nuts, reduce the incidence of major cardiovascular events by 30% within the context of primary prevention [12]. Our results further confirm the important role that olive oil consumption may play, even though other key components of the MedDiet, such as nuts, vegetables, fruits, legumes, fish and wine could also contribute to the observed beneficial effects. In agreement with our results, previous observational studies found an inverse association between olive oil consumption and CVD. Thus, in the EPICOR study conducted in 30,000 Italian women followed for 7.8 years, a 44% reduction in the risk of CHD was observed in those women in the top quartile of total olive oil consumption compared to those in the lowest quartile [4]. Similarly, the hazard ratio of CHD in participants in the top quartile of olive oil consumption in the Spanish EPIC cohort was 0.78 (95% CI: 0.59 to 1.03) compared to the reference, after a 10.4-year follow-up. In this study, the reduction of CHD risk was greater for virgin olive oil than for the common variety [5].

We found a strong relationship between total olive oil consumption and the composite of cardiovascular major events, but when we analyzed separately myocardial infarction and stroke, the associations were non-significant. This could be explained by the lack of statistical power, but, according to our results, two previous case-control studies conducted in an Italian population also reported no associations between olive oil intake and myocardial infarction [20,21].

Our data suggest that total olive oil intake was inversely associated with cardiovascular mortality but not with cancer mortality: each 10 g/d increase in total olive oil consumption is associated with a 16% reduction in cardiovascular mortality. These results are supported by the findings of EPIC-Spain, where a 44% reduction in CVD mortality was found in participants at the top quartile of total olive oil consumption compared to those in the bottom quartile [6] and they can contribute to solving the inconsistencies reported by a recent meta-analysis [9]. In the same study of EPIC-Spain, total olive oil intake was not associated with cancer mortality [6]. However, like our study, this study did not examine specific types of cancer. A recent review of epidemiological studies reported some evidence suggesting that olive oil can decrease the risk of upper digestive and respiratory tract neoplasms, breast cancer and probably cancer in other sites [10]. Therefore, future larger studies or meta-analyses may need to focus on incidence and mortality of specific cancer sites.

The outcomes regarding olive oil consumption and all-cause mortality have been inconsistent, as reported in a recent meta-analysis including a large number of participants [9]. A previous study conducted in participants from Italy, who have suffered a myocardial infarction, showed that there was a 24% (HR: 0.76; 95% CI: 0.64 to 0.91) reduction in the risk of overall mortality for those consuming olive oil regularly compared to those who never consumed olive oil [7]. The results from the EPIC-cohort indicated a 26% (HR: 0.74; 95% CI: 0.64 to 0.87) reduction in the risk of overall mortality for those in the highest quartile of total olive oil intake compared to the lowest quartile [6]. On the contrary, no associations were found between total olive oil intake and all-cause mortality in a free-living Greek population [22]. Our findings suggest a non-significant possible inverse relation between each 10 g/d (one tablespoon of oil) increase in total olive oil and EVOO consumption and all-cause mortality.

The inverse associations between olive oil consumption and CVD could be explained by several mechanisms. The beneficial effects of olive oil are mainly attributed to its high content on MUFAs (that are less susceptible to oxidation than other type of fatty acids) but also to other minor components with important biological properties, such as phenolic compounds, vitamin E and other lipid-derivate molecules (squalene, tocopherols, triterpenic alcohols and so on), especially occurring in EVOO [2,3]. Evidence suggests that olive oil has anti-inflammatory and anti-atherogenic effects and it may also play a beneficial role in reducing oxidative stress and improving endothelial function [3,23,24]. Moreover, it has been observed that EVOO, particularly in a context of a MedDiet, improved lipid profile, insulin sensitivity, glycemic control, decreased blood pressure [11,24-27] and also was inversely associated with new-onset diabetes [28], all of them considered strong risk factors for CVD.

The strengths of the present study are its prospective design and a relatively long duration of follow-up. In addition, previous studies made no distinctions among different varieties of olive oil, but we analyzed the associations for EVOO and common olive oil separately. The present study was conducted in a population where the intake of total olive oil was relatively high, allowing a better assessment of the association between olive oil consumption and CVD or mortality.

Some limitations of our study deserve attention. First, the generalizability of our results may be limited, as the studied population was composed of Mediterranean elderly individuals at high cardiovascular risk who increased their intake of olive oil due to the intervention. However, the findings from our study are broadly consistent with those from other populations. Although the individuals changed their oil consumption during the study and this could have affected the observed beneficial effects of olive oil, it should be noted that the baseline intake of olive oil was high and the baseline assessment can be considered as a good correlate of lifetime habits in this population. Second, because of the observational nature of the study, residual confounding remains a possibility even though our analyses were extensively adjusted for a wide range of cardiovascular risk factors. Nonetheless, our observational findings are consistent with the intervention effects observed in the olive oil enriched arm in the PREDIMED trial. Finally, although the FFQ used was validated, measurement errors are inevitable, especially regarding self-reported different varieties of olive oil.

## Conclusions

In summary, we found that greater consumption of total olive oil, especially EVOO, was associated with reduced cardiovascular disease and mortality risk in an elderly Mediterranean population at high cardiovascular risk. Our findings underscore olive oil consumption as one of the key components of the MedDiet for cardiovascular disease prevention.

## Abbreviations

BMI: Body mass index; CHD: Coronary heart disease; CVD: Cardiovascular disease; EPIC: European Prospective Investigation into Cancer and Nutrition; EVOO: Extra virgin olive oil; FFQ: Food frequency questionnaires; HDL: High-density lipoprotein; LDL: Low-density lipoprotein; MedDiet: Mediterranean diet; MUFA: Monounsaturated fatty acids.

## Competing interests

No potential conflicts of interest relevant to this article were reported by any of the authors.

## Authors’ contributions

MAM-G, RE, ER, DC, EG-G, MF, JL, LS-M, MAM, XP, RML-R, VR-G and JS-S designed the research. MG-F, FBH, MAM-G, MF, MB, RE, ER, DC, JR, EG-G, MF, JL, LS-M, MAM, XP, RML-R, JB, PB-C, JVS, VR-G, JAM and JS-S conducted the research. M-GF, FBH and JS-S analyzed the data. MG-F and JS-S wrote the paper. MAM-G, RE, ER, DC, MF, JL, LS-M, MAM, XP and JS-S were the coordinators of subject recruitment at the outpatient clinics. MG-F and JS-S had full access to all the data in the study and take responsibility for the integrity of the data and the accuracy of the data analysis. All authors revised the manuscript for important intellectual content, and read and approved the final manuscript.

## Pre-publication history

The pre-publication history for this paper can be accessed here:

http://www.biomedcentral.com/1741-7015/12/78/prepub

## Supplementary Material

Additional file 1 **Total olive oil intake during follow-up.** Changes in total olive oil consumption by year during the follow-up for the total participants, and also by intervention group. Click here for file
