# Supplementary material for: Olive oil intake and risk of cardiovascular disease and mortality in the PREDIMED Study
Source: BMC Med. 2014 May 13;12:78. doi: 10.1186/1741-7015-12-78 (PMC4030221; doi:10.1186/1741-7015-12-78)
Supplement: Additional file 1 — Total olive oil intake during follow-up. Changes in total olive oil consumption by year during the follow-up for the total participants, and also by intervention group. [file 1741-7015-12-78-S1.docx]

**File name:** ADDITIONAL FILE 1

**Format:** Word

**Title of data:** Total olive oil intake during follow-up

**Description of data:** Changes in total olive oil consumption by year during the follow-up for the total participants, and also by intervention group.
